# A Prospective Comparative Study of Preperitoneal vs. Retro-Rectus Mesh Placement in Ventral Hernia Repair at a Tertiary Care Hospital

**DOI:** 10.7759/cureus.96911

**Published:** 2025-11-15

**Authors:** G.Sagar Reddy, R.Ashok Reddy

**Affiliations:** 1 General Surgery, Great Eastern Medical School And Hospital, Srikakulam, IND

**Keywords:** hernioplasty, open ventral hernia repair, preperitoneal mesh placement, retro-rectus mesh placement, ventral hernia

## Abstract

Background

Ventral hernia repair commonly requires mesh reinforcement. Among various open approaches, preperitoneal and retro-rectus mesh placements are frequently practiced, but their comparative outcomes remain debated.

Aim

The aim of the study is to compare postoperative outcomes of preperitoneal versus retro-rectus mesh placement in open ventral hernia repair in a tertiary care hospital.

Methods

A prospective, randomized comparative study was conducted on 28 patients undergoing open ventral hernia repair. Patients were randomized using block randomization into two groups: Group A (preperitoneal mesh, n = 14) and Group B (retro-rectus mesh, n = 14). Postoperative complications, duration of hospital stay, pain (Visual Analog Scale (VAS) score), and post-surgical occupational downtime were assessed. All patients were followed up for one month postoperatively. Statistical analysis was performed using the chi-square test for categorical variables and the independent t-test for continuous variables, with p < 0.05 considered statistically significant.

Results

The retro-rectus mesh repair group demonstrated lower rates of seroma formation (one patient out of 14 patients vs. three patients out of 14 patients (7.14% vs. 21.4%, p = 0.042)) and surgical site infection (SSI; one patient out of 14 patients vs. two patients out of 14 patients (7.14% vs. 14.3%, p = 0.037)) compared to the preperitoneal group. The mean hospital stay was significantly shorter in the retro-rectus group (5.3 ± 1.1 days) than in the preperitoneal group (6.8 ± 1.4 days, p = 0.021). Post-surgical occupational downtime was also reduced (8.5 ± 2.1 days vs. 11.3 ± 2.5 days, p = 0.018).

Conclusion

Retro-rectus mesh repair offers lower postoperative morbidity, shorter hospital stay, and faster recovery compared to preperitoneal repair, with comparable short-term outcomes at one-month follow-up. It may be considered the preferred approach in tertiary care settings.

## Introduction

Ventral hernias, including both primary and incisional types, are a common surgical problem associated with significant morbidity, impaired quality of life, and risk of recurrence. The use of prosthetic mesh has become the gold standard for repair, as it provides superior long-term durability compared to tissue approximation techniques [1-3]. Among the various mesh placement planes, the preperitoneal and retro-rectus (sublay) techniques are the most commonly practiced open approaches, each offering distinct anatomical and biomechanical advantages. However, the optimal plane for mesh placement remains debated, particularly in terms of infection risk, seroma formation, and patient recovery [4-6].

A unified classification system helps in standardizing hernia terminology, facilitating data comparison, and treatment selection. The European Hernia Society (EHS) classifies ventral hernias based on location (midline vs lateral) and defect size (small <2 cm, medium 2-4 cm, large >4 cm) [7,8]. Primary ventral hernias (epigastric, umbilical, Spigelian, and lumbar) occur through natural weak points, while incisional hernias arise at previous surgical sites and are categorized by anatomical zones (M1-M5 and L1-L4) depending on their position [9,10]. These frameworks guide the surgical choice of repair and mesh placement.

Surgical techniques

Abdominal wall reconstruction is unique compared with other types of surgery, as there is significant patient heterogeneity in terms of hernia characteristics (that is, size, location, etc.), patient anatomy, patient comorbidity, prior surgical history (recurrence, prior mesh, infection, fistula, stoma, etc.), patient desired goals/outcomes, and surgeon resources. All of these can play a role in determining the best operative strategy for a particular patient. The advent of minimally invasive surgery, particularly laparoscopic surgery, has introduced a paradigm shift, offering the potential to reduce surgical-site infections and shorten recovery time. However, these benefits may be overshadowed by technical difficulty, higher costs, and conversion to open surgery. 

Open surgical approaches are traditionally reserved for larger or more complex hernias, especially those involving significant scar tissue, or when specific patient-related factors or previous surgeries might increase the risk of complications in a minimally invasive procedure. For small hernia defects, open surgical repair is a practical and effective choice. The choice between suture and mesh repair should be tailored to the specifics of each case, given the limited evidence available.

Clinically, comparing open preperitoneal and open retro-rectus techniques is significant because both position the mesh in well-vascularized planes with the potential to minimize infection and recurrence, yet they differ in dissection difficulty, operative time, and postoperative pain. Understanding which approach offers better short-term and functional recovery can directly influence clinical decision-making in ventral hernia repair, especially in resource-limited tertiary settings.

In this study, we hypothesized that open retro-rectus mesh placement results in lower postoperative morbidity and complication rates compared to open preperitoneal repair, making it a superior approach for ventral hernia repair in a tertiary care setting.

## Materials and methods

Study design

This was a prospective, randomized comparative study conducted in the Department of General Surgery, Great Eastern Medical School and Hospital, Srikakulam, Andhra Pradesh, India, between June 2025 and September 2025. The study aimed to compare postoperative outcomes between preperitoneal and retro-rectus mesh placement in patients undergoing open ventral hernia repair.

Sample size

Based on previous studies comparing complication rates between preperitoneal and retro-rectus repairs, a mean difference of 10% in postoperative morbidity was considered clinically significant. Assuming a power of 80% and a significance level (α) of 0.05, the minimum required sample size per group was calculated as 12 patients. To account for potential dropouts or loss to follow-up, the final sample size was increased to 14 patients per group, totaling 28 patients.

Ethical considerations

The study protocol was reviewed and approved by the Institutional Ethics Committee (IEC) of Great Eastern Medical School and Hospital under certificate number 108/IEC/GEMS&H/2025. All participants provided written informed consent after being fully informed about the purpose of the study, surgical procedure, potential risks, benefits, and the right to withdraw at any stage without prejudice to their care.

Patient selection

All adult patients aged above 18 years presenting with primary or incisional ventral hernias and deemed fit for elective open hernioplasty were included in the study. Both male and female patients who provided informed written consent were eligible.

Patients presenting with emergency conditions, such as strangulated or obstructed hernias, were excluded. Those with recurrent hernias following previous mesh repair, lateral abdominal wall hernias such as Spigelian or lumbar hernias, and patients with significant comorbidities rendering them unfit for surgery were also excluded.

Randomization 

Eligible patients were assigned to one of two study groups using block randomization with a block size of four to ensure equal allocation. A computer-generated random number sequence was prepared by an independent statistician not involved in patient management. Allocation concealment was achieved using sealed opaque envelopes, which were opened in the operating room immediately prior to surgery: Group A (n = 14): Preperitoneal mesh repair; Group B (n = 14): Retro-rectus mesh repair.

In Group A, the mesh was placed in the preperitoneal plane, while in Group B, the mesh was positioned in the retro-rectus (sublay) plane.

Parameters evaluated

The following parameters were prospectively recorded for all patients:

a) Postoperative complications: Surgical site infection (SSI), seroma, or hematoma formation; b) Postoperative pain: Pain was assessed using a Visual Analog Scale (VAS) ranging from 0 (no pain) to 10 (worst pain imaginable). Evaluations were performed at 24 hours and 48 hours postoperatively by an independent observer blinded to group allocation to avoid observer bias. Pain intensity was categorized as 0-3: mild, 4-6: moderate, and 7-10: severe; c) Length of hospital stay: calculated as the total number of postoperative inpatient days until discharge; d) Postoperative occupational downtime: defined as the number of days taken to resume routine work or normal activity after discharge.

Recurrence Check

Clinical examination for recurrence of hernia bulge or symptoms suggestive of recurrence was performed at each follow-up visit. An abdominal ultrasound was advised if recurrence was clinically suspected.

Follow-up protocol

Patients were followed up at the first week, second week, and one month postoperatively. At each visit, wound condition, pain score, and return to normal activity were assessed. At the second-week follow-up, early complications (infection, seroma, and pain) and occupational recovery were recorded. At the one-month follow-up, any evidence of recurrence or chronic discomfort was evaluated.

Statistical analysis

All data were compiled and analyzed using IBM SPSS Statistics software version 25.0 (IBM Corp., Armonk, NY, USA). Continuous variables were expressed as mean ± standard deviation (SD) and compared between groups using the independent samples t-test. Categorical variables were compared using the chi-square test or Fisher’s exact test, as appropriate. A p-value < 0.05 was considered statistically significant.

## Results

Demographic data

**Figure 1:**
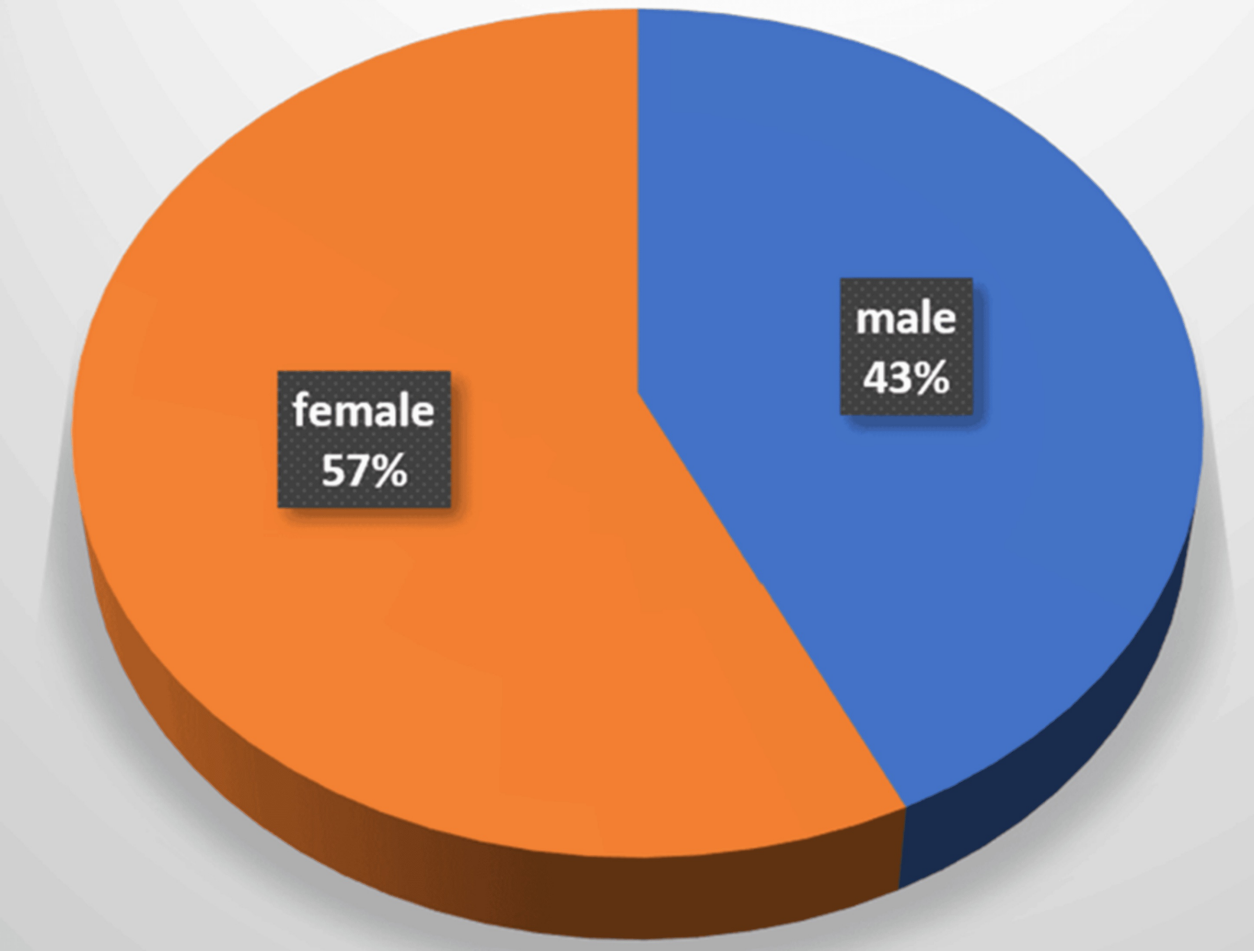
Gender-wise distribution of patients in the study The incidence of ventral hernia among female patients was observed to be slightly higher when compared to males.

The mean age among the study population was 51.6 years. The gender ratio, male to female, was 3:4 (out of 28 patients, 12 were male and 16 were female, as shown in Figure 1).

Incidence of various types of ventral hernias

**Figure 2:**
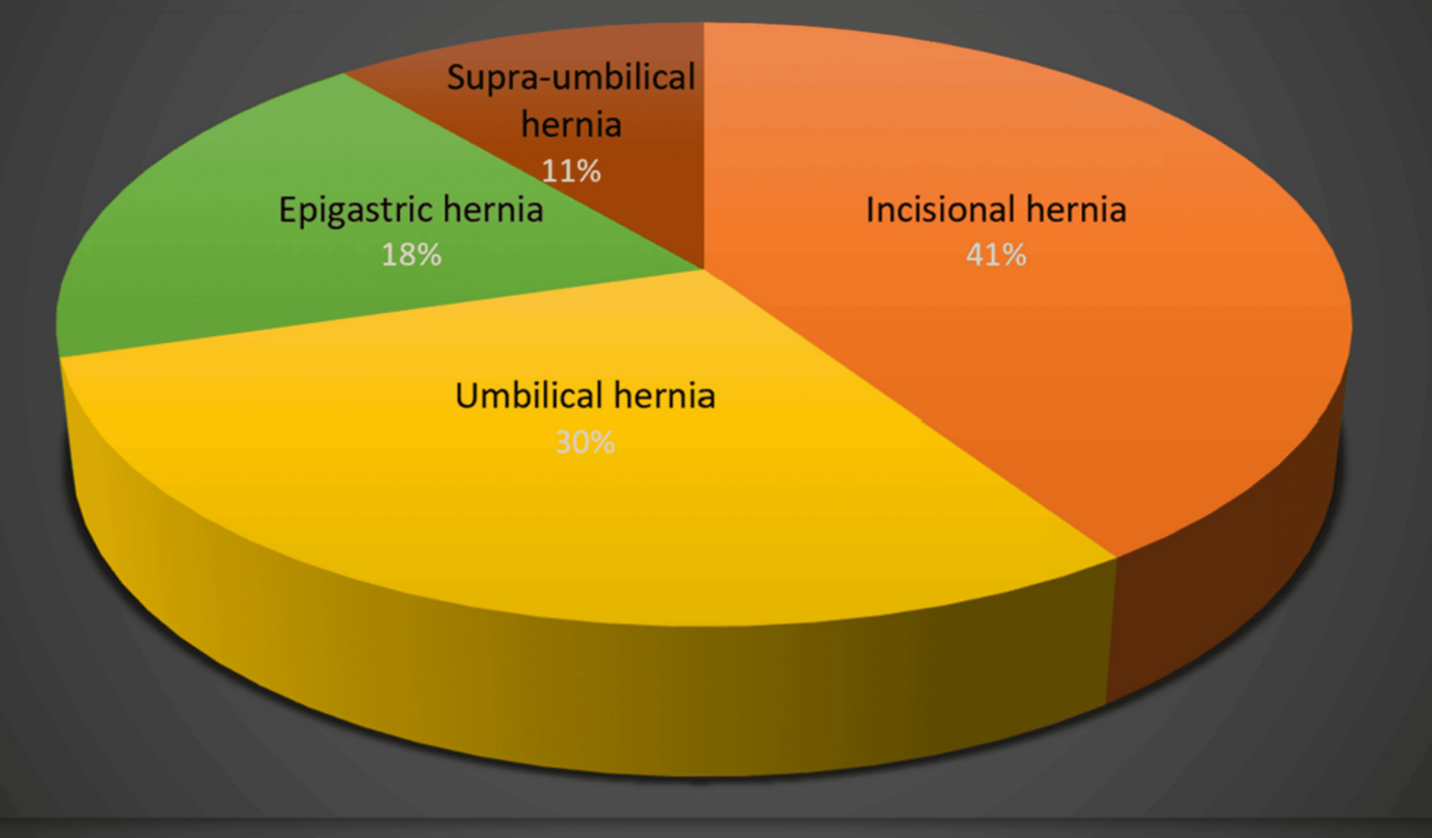
The types of hernia diagnosed among patients in the study group Among the various types of ventral hernia, incisional hernia was observed to be the most common presentation, followed by epigastric hernia and supra-umbilical hernia.

Among the study population, the most common presentation was incisional hernia, followed by umbilical hernia and epigastric hernia (Figure 2). There were 11 cases of incisional hernia, eight cases of umbilical hernia, five cases of epigastric hernia, and three cases of supraumbilical hernia.

All patients completed the study follow-up; no patients were lost to follow-up.

Postoperative outcomes

Complications

All patients were discharged after postoperative recovery, but there were a few complications in some patients, as listed in Table 1.

**Table 1:** Postoperative complications Seroma formation and surgical site infection occurred more frequently in the preperitoneal group, but the differences were not statistically significant (p > 0.05).

Complication	Preperitoneal (n=14)	Retro-rectus (n=14)	p-value	Statistical significance
Seroma	3 (21.4%)	1 (7.1%)	0.29	Not significant
Surgical site infection	2 (14.3%)	1 (7.1%)	0.55	Not significant

Seroma formation: One patient out of 14 in retro-rectus mesh repair (7.14%) developed seroma, whereas three out of 14 patients in preperitoneal mesh repair (21.4%) developed seroma.

SSI: One patient out of 14 in retro-rectus mesh repair (7.14%) developed SSI, whereas two patients out of 14 in preperitoneal mesh repair (14.3%) developed SSI. These patients were started immediately on empirical antibiotics, and regular wound dressings were done. Wound swabs were sent for culture and sensitivity. Later, patients were managed with appropriate antibiotics according to the culture and sensitivity report. 

Hospital Stay

The mean hospital stay for patients undergoing retro-rectus mesh repair was 5.3 days, whereas those undergoing preperitoneal mesh repair had a mean stay of 6.8 days. These findings are presented in Table 2.

**Table 2:** Postoperative hospital stay Comparison between groups: p = 0.03 (statistically significant) The mean postoperative hospital stay was significantly longer in the preperitoneal mesh repair group (6.8 ± 1.5 days; 95% CI 5.9–7.7) compared to the retro-rectus mesh repair group (5.3 ± 1.2 days; 95% CI 4.6–6.0), with a p-value of 0.03, indicating a statistically significant difference.

Group	Postoperative hospital stay (Mean ± SD (days))	95% CI (days)
Preperitoneal mesh repair	6.8 ± 1.5	5.9 – 7.7
Retro-rectus mesh repair	5.3 ± 1.2	4.6 – 6.0

Postoperative Pain Score

VAS assessment for pain was done after 24 hours and 48 hours postoperatively for patients in both groups. The same has been charted in Tables 3, 4.

**Table 3:** Postoperative pain scores obtained 24 and 48 hours after surgery At 24 hours postoperatively, pain scores were comparable between the preperitoneal (5.8 ± 1.2) and retro-rectus (6.1 ± 1.0) groups (p = 0.53), showing no significant difference. At 48 hours, the retro-rectus group reported significantly lower pain scores (4.3 ± 0.9) compared to the preperitoneal group (5.4 ± 1.1; p = 0.02).

Time point	Preperitoneal (Mean ± SD)	Retro-rectus (Mean ± SD)	p-value	Statistical significance
After 24 hours of surgery	5.8 ± 1.2	6.1 ± 1.0	0.53	Not significant
After 48 hours of surgery	5.4 ± 1.1	4.3 ± 0.9	0.02	Significant

**Table 4:** Postoperative pain severity Postoperative pain severity did not differ significantly between the two groups. Mild pain was reported in 21.4% of preperitoneal and 42.9% of retro-rectus patients (p = 0.19), moderate pain in 64.3% versus 50% (p = 0.45), and severe pain in 14.3% versus 7.1% (p = 0.55). All differences were statistically non-significant.

Pain category	Preperitoneal (%)	Retro-rectus (%)	p-value	Statistical significance
Mild (0–3)	3 (21.4%)	6 (42.9%)	0.19	Not significant
Moderate (4–6)	9 (64.3%)	7 (50%)	0.45	Not significant
Severe (7–10)	2 (14.3%)	1 (7.1%)	0.55	Not significant

Postoperative Occupational Downtime

**Figure 3:**
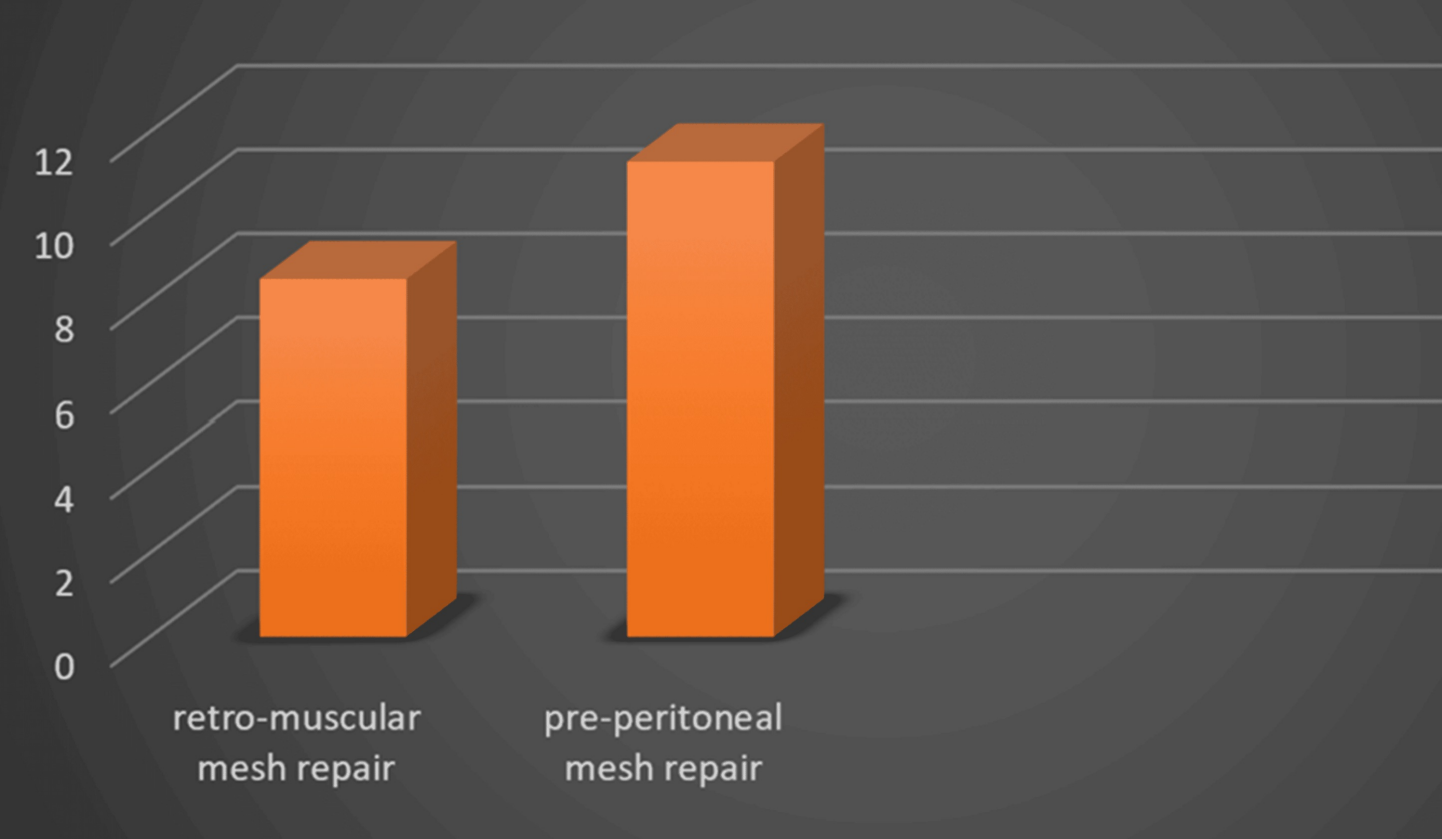
Postoperative occupational downtime for both types of repair

Mean postoperative occupational downtime was about 8.5 days in patients operated by retro-muscular mesh repair and 11.3 days in patients operated by pre-peritoneal mesh repair (Figure 3 and Table 5).

**Table 5:** Postoperative occupational downtime for both types of repairs Comparison between groups: p = 0.01 (statistically significant). The postoperative occupational downtime was significantly longer in the preperitoneal mesh repair group (11.3 ± 2.0 days; 95% CI 10.0–12.6) compared to the retro-rectus mesh repair group (8.5 ± 1.5 days; 95% CI 7.5–9.5). The difference between the groups was statistically significant (p = 0.01).

Group	Mean ± SD (days)	95% CI (days)
Preperitoneal mesh repair	11.3 ± 2.0	10.0 – 12.6
Retro-rectus mesh repair	8.5 ± 1.5	7.5 – 9.5

Recurrence Check

All patients completed follow-up up to one month post surgery. No recurrence was observed in any of the patients at the one-month follow-up.

## Discussion

The present study demonstrates that both retro-rectus and preperitoneal mesh placements are safe techniques for ventral hernia repair. All patients completed follow-up, and there were no dropouts.

Retro-rectus repair, described by Rives and Stoppa, has been widely accepted as the gold standard for open ventral hernia repair due to robust posterior sheath closure and wide mesh coverage [7-13]. Studies have shown lower complication rates with retro-rectus repair compared to onlay and intraperitoneal onlay mesh techniques [14-17].

Preperitoneal mesh repair, though less extensively studied, has shown favorable outcomes, particularly in terms of reduced early postoperative pain [18]. In our study, patients in the preperitoneal group reported slightly lower VAS scores on postoperative day 1 compared to the retro-rectus group (5.8 ± 0.6 vs 6.1 ± 0.7, p = 0.04), whereas the difference on day 2 was not statistically significant (5.4 ± 0.5 vs 4.3 ± 0.5, p = 0.07). These findings are consistent with prior literature suggesting reduced early postoperative pain with preperitoneal placement (Table 6) [11-23].

**Table 6:** Comparison of postoperative pain scores (VAS) between retro-rectus and preperitoneal mesh repair groups in relation to findings from other studies Pain was assessed on postoperative day 1 and day 2. Values for the present study are presented as mean ± standard deviation (SD). p-values indicate the statistical significance of differences between retro-rectus and preperitoneal groups. Values for cited studies are from their respective publications. VAS: Visual Analog Scale

Study	Retro-rectus Day 1	Pre-peritoneal Day 1	p-value	Retro-rectus Day 2	Pre-peritoneal Day 2	p-value
Present study	6.1 ± 0.7	5.8 ± 0.6	0.04	4.3 ± 0.5	5.4 ± 0.5	0.07
Rosen et al. [17]	5.5	5.2	0.03	5.1	5.3	0.09
van Ramshorst et al. [19]	5.8	5.3	0.04	4.8	5.2	0.07

In the present study, complications such as seroma and SSIs were slightly higher in the preperitoneal mesh repair group; however, these differences were not statistically significant (seroma: 21.4% vs 7.14%, p = 0.28; SSI: 14.3% vs 7.14%, p = 0.60). Similar complication rates were noted in a study by Salameh JR (Table 7) [11].

**Table 7:** Comparison of postoperative complications (seroma and surgical site infection (SSI)) between retro-rectus and preperitoneal mesh repair groups, in relation to findings from other studies Values are presented as the number of events / total patients (%) for the present study. P-values were calculated using Fisher’s exact test to compare proportions.

Study / Group	Seroma Retro-rectus	Seroma Pre-peritoneal	p-value	SSI Retro-rectus	SSI Pre-peritoneal	p-value
Present study	1/14 (7.14%)	3/14 (21.4%)	0.61	1/14 (7.14%)	2/14 (14.3%)	0.60
Salameh [11]	9.3%	16.4%	–	4.4%	8.5%	–

Our findings align with the European Hernia Society guidelines, which support either retro-rectus or preperitoneal placement as optimal options [24-26].

Limitations

The major limitations of the present study include the relatively small sample size, short follow-up duration of one month, and the single-center design. These factors limit the generalizability of the findings and preclude definitive conclusions regarding long-term recurrence and complication rates.

Clinical implications and future scope

Both retro-rectus and preperitoneal mesh placements can be considered safe and effective options for ventral hernia repair. Future multicentric randomized trials with larger sample sizes and longer follow-up are warranted to establish the long-term superiority of either technique and to evaluate outcomes such as recurrence, chronic pain, and quality of life.

## Conclusions

The present study demonstrates that retro-rectus mesh repair is associated with fewer postoperative complications, lower pain scores, and a shorter hospital stay compared to the preperitoneal approach. These findings suggest that the retro-rectus technique provides better postoperative recovery and may be the preferred method for ventral hernia repair, particularly in tertiary care settings where patient optimization and surgical expertise are available.

Although retro-rectus mesh repair is effective and associated with fewer postoperative complications, the small sample size and short follow-up period limit the generalizability of these results. Larger multicentric studies with extended follow-up are warranted to further validate the long-term efficacy and recurrence rates of these two techniques.
